# Evaluation of two vaginal, uterus sparing operations for pelvic organ prolapse: modified Manchester operation (MM) and sacrospinous hysteropexy (SSH), a study protocol for a multicentre randomized non-inferiority trial (the SAM study)

**DOI:** 10.1186/s12905-019-0749-7

**Published:** 2019-04-02

**Authors:** Sascha F. M. Schulten, Rosa A. Enklaar, Kirsten B. Kluivers, Sanne A. L. van Leijsen, Marijke C. Jansen-van der Weide, Eddy M. M. Adang, Jeroen van Bavel, Heleen van Dongen, Maaike B. E. Gerritse, Iris van Gestel, G. G. Alec Malmberg, Ronald J. C. Mouw, Deliana A. van Rumpt-van de Geest, Wilbert A. Spaans, Annemarie van der Steen, Jelle Stekelenburg, E. Stella M. Tiersma, Anneke C. Verkleij-Hagoort, Astrid Vollebregt, Chantal B. M. Wingen, Mirjam Weemhoff, Hugo W. F. van Eijndhoven

**Affiliations:** 1 Department of Obstetrics and Gynaecology, Radboud university medical center, Geert Grooteplein Zuid 10, 6525 GA Nijmegen, The Netherlands; 2 Department of Obstetrics and Gynaecology, Isala Zwolle, Dokter van Heesweg 2, 8025 AB Zwolle, The Netherlands; 3 Department of Obstetrics and Gynecology, Zuyderland Medical Center, Henri Dunantstraat 5, 6419, PC Heerlen, The Netherlands; 4 Department of Obstetrics and Gynaecology, Máxima Medical Centre Veldhoven, De Run, 4600 5500, MB Veldhoven, The Netherlands; 5 Department of Obstetrics and Gynaecology, Amsterdam University Medical Centre, University of Amsterdam, Meibergdreef 9, 1105 AZ Amsterdam, The Netherlands; 6 Department for Health Evidence, Radboud university medical center, Geert Grooteplein 27, 6525 EZ, Nijmegen, The Netherlands; 7 Department of Obstetrics and Gynaecology Amphia Hospital, Molengracht 21, 4818 CK Breda, The Netherlands; 8 Department of Obstetrics and Gynaecology Groene Hart Hospital, Bleulandweg 10, 2803 HH Gouda, The Netherlands; 9 Department of Obstetrics and Gynaecology Gelderse Vallei Hospital, Willy Brandtlaan 10, 6716 RP Ede, The Netherlands; 10 Department of Obstetrics and Gynaecology, Viecuri Hospital, Tegelseweg 210, 5912 BL Venlo, The Netherlands; 11 Department of Obstetrics and Gynaecology, University Medical Centre Groningen, University of Groningen, Hanzeplein 1, 9713 GZ Groningen, The Netherlands; 12 Department of Obstetrics and Gynaecology, Rijnstate Hospital, Wagnerlaan 55, 6815 AD Arnhem, The Netherlands; 13 Department of Obstetrics and Gynaecology, Reinier de Graaf Hospital, Reinier de Graafweg 5, 8934 AD Delft, The Netherlands; 14 Department of Obstetrics and Gynaecology, Maastricht University Medical Centre, P. Debyelaan 25, 6229 HX, Maastricht, The Netherlands; 15 Department of Obstetrics and Gynaecology, Ziekenhuisgroep Twente, Zilvermeeuw 1, 7609 PP Almelo, The Netherlands; 16 Department of obstetrics and Gynaeology, Medisch Spectrum Twente, Koningstraat 1, 7512 KZ Enschede, The Netherlands; 17 Department of Obstetrics and Gynaecology, Medical Centre Leeuwarden, Henri Dunantweg 2, 8934 AD Leeuwarden, The Netherlands; 18 Department of Obstetrics and Gynaecology, st. Antonius hospital, Koekoekslaan 1, 3435 CM Nieuwegein, The Netherlands; 19 Department of Obstetrics and Gynaecology, Spaarne Gasthuis, Spaarnepoort 1, 2134 TM Hoofddorp, The Netherlands; 20 Department of Obstetrics and Gynaecology, Laurentius Hospital, Monseigneur Driessenstraat 6, 6043 CV Roermond, The Netherlands; 21 Radboud university medical center, Department of Obstetrics and Gynaecology, PO Box 9101, 6500 HB Nijmegen, The Netherlands

**Keywords:** Sacrospinous hysteropexy, Modified Manchester operation, Uterine descent, Pelvic organ prolapse, POP-Q, Reconstructive surgery, Randomized clinical trial, Cost-utility

## Abstract

**Background:**

Pelvic organ prolapse (POP) affects up to 40% of parous women which adversely affects the quality of life. During a life time, 20% of all women will undergo an operation. In general the guidelines advise a vaginal operation in case of uterine descent: hysterectomy with uterosacral ligament plication (VH), sacrospinous hysteropexy (SSH) or a modified Manchester operation (MM). In the last decade, renewed interest in uterus sparing techniques has been observed. Previous studies have shown non-inferiority between SSH and VH. Whether or not SSH and MM are comparable concerning anatomical and functional outcome is still unknown. The practical application of both operations is at least in The Netherlands a known cause of practice pattern variation (PPV). To reveal any difference between both techniques the SAM-study was designed.

**Methods:**

The SAM-study is a randomized controlled multicentre non-inferiority study which compares SSH and MM. Women with symptomatic POP in any stage, uterine descent and POP-Quantification (POP-Q) point D at ≤ minus 1 cm are eligible. The primary outcome is the composite outcome at two years of absence of prolapse beyond the hymen in any compartment, the absence of bulge symptoms and absence of reoperation for pelvic organ prolapse. Secondary outcomes are hospital parameters, surgery related morbidity/complications, pain perception, further treatments for prolapse or urinary incontinence, POP-Q anatomy in all compartments, quality-of-life, sexual function, and cost-effectiveness. Follow-up takes place at 6 weeks, 12 and 24 months. Additionally at 12 weeks, 6 and 9 months cost-effectiveness will be assessed. Validated questionnaires will be used and gynaecological examination will be performed. Analysis will be performed following the intention-to-treat and per protocol principle. With a non-inferiority margin of 9% and an expected loss to follow-up of 10%, 424 women will be needed to prove non-inferiority with a confidence interval of 95%.

**Discussion:**

This study will evaluate the effectiveness and costs of SSH versus MM in women with primary POP. The evidence will show whether the existing PPV is detrimental and a de-implementation process regarding one of the operations is needed.

**Trial registration:**

Dutch Trial Register (NTR 6978, http://www.trialregister.nl). Date of registration: 29 January 2018. Prospectively registered.

## Background

Pelvic organ prolapse (POP) is a frequently diagnosed health problem in women which negatively affects the quality of life. 40% of vaginally parous women have at least stage 2 POP [1, 2]. In the Netherlands, 20% of all women will undergo POP surgery during life time [3, 4]. According to the current guidelines, the first choice surgical treatment is a vaginal POP operation [5]. When undergoing surgery these women have a 5–30% risk on re-operation due to recurrence [2, 6]. According to a nationwide Dutch registration (Kiwa Carity), 15,000 vaginally POP procedures are performed each year in the Netherlands [7]. Currently, three types of vaginal operations for uterine descent are performed the most; the vaginal hysterectomy (VH), the modified Manchester operation (MM) and the sacrospinous hysteropexy (SSH), all combined with colporrhaphy when indicated. SSH and MM are uterus preserving techniques in which the uterus is attached to different pelvic ligaments. The number of uterus sparing operations (SSH and MM) has increased more than fivefold between 1997 and 2009 in the Netherlands, at the expense of VH [8]. Still the three surgical treatments co-exist without solid evidence-based literature on comparison, which leads to practice pattern variation (PPV) between hospitals and gynaecologists. PPV is defined as a difference in care that cannot be explained by the underlying medical condition [9]. For example, in the Netherlands choice of treatment is more or less equally divided between the three procedures for women with stage two uterine descent (varying from 25 to 34%) [7, 8]. This indicates a situation of maximal practice pattern variation. The choice of treatment is dictated by the physician’s preference or skills and is (probably) not evidence based. Theoretically, PPV can lead to increased health costs. Additionally, the lack of evidence makes it more difficult to come to a process of shared decision making between doctor and patient and individually tailored management in this field.

First steps have been made in filling the knowledge gap; the comparison VH versus SSH has been made in several studies [10–12], and was well established by the SAVE-U randomized multicentre trial [13]. The latter showed equal effectiveness, but shorter operation times in SSH. In case of patient’s preference for uterus preservation [14], the SAVE-U publications have provided arguments to choose for uterus sparing surgery. Recently published systematic reviews by Kapoor et al. and Meriwether et al. confirmed these findings and additionally less blood loss was reported favouring uterus sparing surgery [15, 16]. Unlike these results, the systematic review by de Oliveira et al. showed a lower reoperation rate after VH. However, this review focused on more advanced uterine prolapse (at least stage II, POP-Q point C > 1 cm) [17].

The Manchester operation was first described in 1888 and has since gained ground in urogynaecology. Originally, the operation was a combination of a cervix amputation with anterior colporrhaphy [18]. Later, the cardinal ligaments were plicated and attached to the cervical stump and today the modern version of the operation is applied in which the sacro-uterine ligaments are plicated as well. Despite the scarcity in studies comparing the MM to other operating techniques, a recent historical cohort study by Tolstrup et al. showed superiority of MM compared to VH [19]. In addition, a recent prospective cohort study showed that the MM provides adequate mid-compartment support and gives excellent subjective results after one-year follow up [20].

It is thought that in case of an elongated cervix, MM may be more suitable than SSH. On the other hand, MM may lead to cervical stenosis (menstrual obstruction, poor access for diagnostics) which was reported up to 11% [21]. At the same time, previous studies have shown that SSH can be performed with a reduced operating time of approximately 19 min compared to MM (SSH 59 ± 13 min vs. MM 78 ± 28 min) [22, 23].

Uterine descent stage 3 is generally not treated with MM (but with VH or SSH), which was also seen in a cohort study on MM [24]. Although MM is performed approximately 3000 times a year in the Netherlands, still little is known about the effectiveness compared to SSH.

To our knowledge, most publications on MM and SSH for treatment of POP are based on observational studies. Only one recently performed randomized clinical trial compared the techniques, but contradictive to our design, women with uterine prolapse all underwent hysterectomy [25]. A randomized clinical trial comparing the SSH and MM with preservation of the uterus has not yet been performed. The PPV, women’s preference and upcoming trend towards uterus preserving surgery makes it inevitable to further fill the knowledge gap. This study will provide answer to the question: Is SSH non-inferior to MM in women with signs and symptoms of POP?

## Methods/design

### Study objectives and hypothesis

The objective of this study is to compare the effectiveness of SSH and MM in the treatment of uterine prolapse with POP-Q point D ≤ minus 1 cm. Comparison will be made concerning anatomy, symptoms, re-interventions, POP-recurrence, complications, hospital parameters, pelvic floor dysfunction, quality of life and costs with a follow-up of two years. Based on the current literature, it is hypothesized that both techniques are comparable regarding effectiveness, that is to say the composite outcome of recurrent anatomical POP with complaints or reoperation or pessary therapy.

### Study design

The SAM study is a multicentre, prospective, randomized, open-label clinical trial aimed at assessing the non-inferiority of the effectiveness of SSH compared to MM. This protocol was developed according to the Standardized Protocol Interventions: Recommendations for Interventional Trials (SPIRIT) 2013 statement [26].

### Study population, recruitment and consent

Women aged 18 or older who are eligible for their first surgical treatment for symptomatic pelvic organ prolapse in any stage and with uterine descent and POP-Q point D at ≤ minus 1 cm will be eligible for the study. We do not consider MM to be the appropriate treatment for women with POP-Q point D > minus 1 cm, since we believe that beyond that point the sacro-uterine ligaments will not provide substantial support. In that case, the patient would benefit more from an SSH. Patients with concomitant anterior or posterior wall prolapse will be included.

Women who underwent previous prolapse or other pelvic floor surgery, need concomitant mid-urethral sling surgery, have a wish or need for uterus removal, have a contraindication for uterus preservation (i.e. abnormal endometrial bleeding, endometrial or cervical malignancy), have a future wish for childbearing, have inadequate skills in the Dutch language or are not capable of filling in questionnaires will be excluded from the study. In case indicated, a pap-smear and/or pipelle endometrial biopsy must be normal before inclusion.

Participating centres will be 27 Dutch university, teaching and non-teaching hospitals (a list of participating centres is available at: http://zorgevaluatienederland.nl/sam). The SAM study is affiliated with the Dutch Consortium for Healthcare Evaluation and Research in Obstetrics and Gynaecology, which gives national attention and therefore ensures the right amount of participating hospitals to achieve adequate patient enrolment. Gynaecologists and trainees of all participating hospitals will perform the eligibility assessment. Eligible patients will be counselled about the study, the two operating techniques and follow-up. All patients will be provided written patient information which contains information about the aim of the study, follow-up, advantages and disadvantages. Patients will be given a week to make their decision on participation in the study. Women who agree to participate will be asked to sign written informed consent. The informed consent form will be obtained and signed by the investigator before randomisation. The rules of Good Clinical Practice will be applied.

All eligible patients who are counselled will be registered during inclusion period. From patients who do not want to participate, only reasons for refusal will be registered. All serious adverse events will be reported to the Medical Ethics Committee. Patient enrolment en recruitment is currently ongoing. 3 July 2018 the first patient enrolled the study. Enrolment is expected to conclude August 2020.

### Intervention

SSH: after opening the posterior vaginal wall, the pararectal space is explored at the right side and the sacrospinous ligament is identified. The posterior side of the cervix is attached to the sacrospinous ligament with two non-absorbable size 1 or 0 sutures at least 2 cm medial of the ischial spine. Either this procedure is performed open or using the Capio suturing device.

MM: the procedure consists of extraperitoneal plication of the uterosacral ligaments (and cardinal ligaments where possible) with use of three or four absorbable size 1 sutures and amputation of the cervix. The most cranial suture is fixated through the posterior fornix of the vagina.

In view of substantial variation in operative skills and experience there will be attention to avoid the measurement of learning curve effects in the study. Only centres where both techniques are performed before the start of the study or where mentoring/supervision is organised can participate in the study. All participating gynaecologists must have performed at least one hundred POP procedures, twenty procedures of each technique of which ten performed in the last three years before the beginning of the study. Before the study started, all participating gynaecologists were invited to a master class to discuss all steps of both procedures, to minimize practice variation. Agreement was reached by all participating gynaecologists. One uniform operation report for each procedure was written after these master classes and is used during the study. Only a few steps of the operation will be left to the preference of the gynaecologist, namely: application and type of hydrodissection, manner of cervical amputation and use of scalpel or scissors for opening the vaginal wall. All these steps will be registered. Allocated interventions will only be modified or discontinued in case the operating gynaecologist believes this is necessary during operation. This will be registered.

Anaesthesia will be either general or spinal, depending on the anaesthesiologist and patient preferences. Antibiotics will be given peri-operatively according to the hospital protocol. Bladder catheterisation will be done before operating one-off or with an indwelling catheter. After operation an indwelling catheter must remain in the bladder until the next morning. Urinary retention after removal will be reported. For both procedures anterior and/or posterior colporrhaphy will be performed as indicated.

### Outcome measures

The primary outcome will be success after two years follow-up. Success is defined as the absence of POP beyond the hymen in any compartment (POP-Q, gynaecological examination), the absence of bulge symptoms (absence of bulge symptoms is defined as a negative response to the question, “Do you see or feel a bulge in the vaginal area” (UDI domain genital prolapse score: 0)), and absence of reoperation or pessary therapy for POP [27].

Secondary outcomes of the study will be clinical parameters (surgery time, hospitalisation time), surgery related morbidity/complications (including menstrual problems, hematometra, any problems with uterine access such as diagnostic cervical or endometrial sampling or IUD insertion), pain perception, further treatments for POP or urinary incontinence, anatomy in all compartments using POPQ [28], general quality of life, disease specific quality of life regarding symptoms and impact of symptoms, sexual function, and costs.

### Randomization

After giving informed consent, patients will be randomized in a 1:1 ratio to either MM or SSH, with use of dynamic block randomisation with blocks of 2, 4 or 6. Randomisation will be done by gynaecologists or research nurses using the online software Castor (version 2018.3.11, Castor Electronic Data Capture, Amsterdam, The Netherlands). Due to the nature of the investigational treatments, blinding is not possible. Participating gynaecologists and investigators will not be able to access the randomization sequence. All analyses will be performed in a blinded fashion.

### Sample size calculation

The sample size calculation was based on the expected comparability between the two techniques regarding the success composite of recurrent signs and symptoms of POP after two-years follow-up. We expect a success rate of 89% for both SSH [13] and MM, two years after the intervention. The actual treatment group proportion (SSH) was set at 89% with a non-inferiority margin of 9%, assuming SSH to be below 80% under the null hypothesis of inferiority. The 9% non-inferiority margin was motivated by the margins used in two other large national studies SAVE-U (7%) [13] and PEOPLE study (10%) [29]. Based on a power of 80% and the significance level of the test (α) targeted at 0.025, sample sizes of 193 per group need to be included in the study. With an expected loss to follow-up of 10% [27], a total of 424 women are needed. The sample size calculation was performed with PASS (version 15.0.7, NCSS statistical software, Kaysville, USA).

### Data collection

All women will undergo gynaecological examination including POP-Q which must be done in 45 degrees supine position. All measurements performed during pre-operative and follow-up outpatients visits, as well as the operation report, will be systematically recorded in an electronic Case Report Form in Castor. All data will be kept anonymous where possible. All participants will be assigned an identification code based on number of the hospital and number of inclusion. A list linking the code to the subject will be kept safe by the local investigators. Personal data will be stored for a maximum of 15 years in participating centres.After randomization patients will be sent an e-mail with a link to the pre-operative questionnaires in Castor. If the subject prefers paper questionnaires, written informed consent is needed to get insight in the postal address of the subject. Both groups will fill in the same questionnaires. The pre-operative questionnaire consists of questions on baseline characteristics, and standardized, validated questionnaires about quality of life (EQ5D-5 L: EuroQol-5D-5 L) [30], disease specific quality of life regarding symptoms (Pelvic Floor Disability Index-20) and the impact of those symptoms (Pelvic Floor Impact Questionnaire-7) [31], sexual function (Pelvic organ prolapse Incontinence Sexual Questionnaire-IR) [32], and the validated cost utility questionnaires of the institute for Medical Technology Assessment (iMTA) (PCQ: Productivity Cost questionnaire and MCQ: Medical Consumption Questionnaire) [33]. After surgery, the patients will visit the hospital at 6 weeks, 12 months and 24 months (see Table 1). During the follow-up specific history taking and gynaecological examination including POP-Q will be done. The POP-Q at 12 and 24 months will be performed by another researcher than the operating gynaecologist. Reimbursement of travel expenses is available to minimize no shows. At 6 months, 12 months and 24 months patients will receive abovementioned questionnaires and an additional disease specific questionnaire (Patient Global Impression of Improvement). At 12 weeks and 9 months patients receive the PCQ, MCQ and EQ5D-5 L; these extra moments are incorporated to prevent recall bias. Reminders will be sent after two weeks in case of non-completion of questionnaires. The duration of the follow-up period is set at 2 years because the majority of recurrences after POP surgery will occur in these first two years [34, 35]. Women will be asked for permission to ask them to participate in a longer follow-up study in case this seems valuable after the two year analysis.

**Table 1:** Schematic overview of follow-up

Measurement	Follow-up moment						
	Pre-op	6 wks	12 wks	6 months	9 months	12 months	24 months
Outpatient visit including POP-Q		x				x	x
Baseline characteristics	x						
EQ5D-5 L	x		x	x	x	x	x
PDFI-20, PGI-I, PFIQ-7, PISQ-IR	x^a^			x		x	x
PCQ and MCQ			x	x	x	x	x

^a^without PGI-I

## Statistical analysis

### Data analysis

Regarding the primary outcome, the null hypothesis entails that the success rate of SSH is inferior by a margin of 9% compared to MM. If the lower limit of the 95% confidence interval does not exceed the margin of − 9%, the null hypothesis is rejected, and we will consider SSH to be non-inferior to MM. The statistical analysis will be performed both by the intention to treat (ITT) and per protocol (PP) principle. This means that non-inferiority has to be demonstrated in both the ITT and PP analysis to declare non-inferiority of SSH compared to MM. The treatment effect will be expressed as relative risk with a 95% confidence interval.

For other (secondary) outcomes, summaries of continuous data will be presented as mean ± standard deviation or median and (interquartile) range depending on their distribution. Categorical data will be presented as frequencies. When appropriate, differences between groups will be analysed using the Student’s t test or Mann-Whitney test for continuous data. Comparisons with categorical data will be analyzed using the Chi-square test of the Fisher’s exact test.

### Other study parameters

Subgroup analyses are planned to investigate the effects of the POP surgery with MM versus SSH in the following pre-specified subgroups: age, menopausal status, sexual activity, POP-Q stage, cervical elongation, concomitant vaginal repair and symptoms.

A prognostic marker analysis will be performed to assess which baseline characteristics of the women have prognostic value and/or can be used as treatment selection markers. Missing covariates will be imputed as appropriate (with solid logical strategy). We will use multiple imputation based on other baseline variables that are associated with higher or lower values of the variable that is imputed. We will do sensitivity analyses to evaluate the difference between imputed and non-imputed variables. All analyses will be performed with IBM SPSS Statistics (version 25, Armonk, New York, United States).

### Economic evaluation

The economic evaluation will be performed along-side the clinical trial, which will compare the cost-effectiveness up to 12 months after surgery from a societal perspective. The design of the economic evaluation follows the principles of a cost-utility analysis and adheres to the Dutch guideline for performing economic evaluations in health care [36]. Costs and effects will be measured on a per patient basis. The cost analysis exists of two main parts. On patient level, the costs made within healthcare, costs made by patients/family and costs in other sectors than healthcare will be included. Second, per item of health care consumption standard cost-prices will be determined using the guideline for performing economic evaluations [36]. If standardized prices are not available, full cost prices will be determined via activity-based costing. Productivity losses for patients will be assessed using the iMTA PCQ. The friction-cost method will be applied and in addition, travel time to the hospital and related costs will be considered. It is anticipated that SSH is as effective but significantly cheaper (less operating time) than MM in this target population. If the results confirm this, a cost-minimization analysis will be the method of analysis. Cost will be analysed using a generalized linear model approach with a gamma distribution using a log link to account for possible skewness of the cost data. The effect analysis measures, on previous mentioned follow-up moments, the clinical trial Health Related Quality of Life using de EQ5D-5 L.

Additionally, a budget impact analysis will be performed for consequences of implementation of SSH substituting for MM in the Dutch health care system in the short-to-medium term from the budget holders perspective [37]. The economic analyses will be presented as a separate study.

## Implementability

The current usual care can best be described as: large PPV regarding vaginal POP surgery in women who undergo their first POP operation. The study results should change this usual care in the following way: implementation of the most cost-effective technique of uterus sparing vaginal POP operation. To increase the generalizability and the implementability of the results, the study will be performed according to high standards in those centres with research facilities of the NVOG Consortium 2.0. Representatives of most target groups are thus involved as including centre or as members of the project group. It is known that implementation is extremely facilitated by involvement in evidence collection [38]. Moreover, these results will be translated into specific and clear recommendations for national clinical guidelines, which will be incorporated in the guideline facilitated by the Dutch Society for Obstetricians and Gynaecologists (NVOG). In addition, the following activities will be performed to transfer our knowledge: study results will be published in an international journal, study results will be disseminated among participants and opinion leaders, results will be incorporated in education programs and patient organizations will distribute the results to their respective platforms and patient information leaflets will be updated.

To optimize implementation of our study results we will focus on potential barriers and facilitators from the start of the study [38]. For that purpose, we will perform qualitative research among the final target users (patients and clinical professionals (e.g. participants and non-participants of this proposed study).

## Discussion

This is a protocol for a randomized clinical trial comparing SSH with MM. To our knowledge this will be the first large randomized study with mid-term follow-up that will compare these two uterus sparing operative techniques. The study will provide knowledge that can lead the way in the best individual treatment in women with POP, can be used to supplement the current guidelines and may show the need for de-implementation of one of the procedures. The study will reveal whether the existing PPV is unwanted or not significant in terms of effectiveness and costs. In case non-inferiority is found, the secondary outcomes might determine the preferred uterus preserving technique.

## Abbreviations

MM: Modified Manchester operation
POP: Pelvic organ prolapse
SSH: Sacrospinous hysteropexy
VH: Vaginal hysterectomy
